# Low HDL levels in sepsis versus trauma patients in intensive care unit

**DOI:** 10.1186/s13613-017-0284-3

**Published:** 2017-06-06

**Authors:** Sébastien Tanaka, Julien Labreuche, Elodie Drumez, Anatole Harrois, Sophie Hamada, Bernard Vigué, David Couret, Jacques Duranteau, Olivier Meilhac

**Affiliations:** 1 Assistance Publique des Hopitaux de Paris, Service d’Anesthésie-Réanimation, Hôpitaux Universitaires Paris-Sud, Université Paris-Sud, Hôpital de Bicêtre, le Kremlin-Bicêtre, France; 2 Département de biostatistique, Université de Lille, CHU Lille, EA 2694 - Santé publique, épidémiologie et qualité des soins, 59000 Lille, France; 3 Laboratoire d’étude de la Microcirculation, « Bio-CANVAS: biomarqueurs in CardioNeuroVascular DISEASES », UMRS 942, Paris, France; 4 Université de La Réunion, INSERM, UMR 1188 Diabète athérothombose Réunion Océan Indien (DéTROI), Saint-Denis, France; 5 CHU de La Réunion, Saint-Denis, France

**Keywords:** Sepsis, Trauma, Lipids, Inflammation, High-density lipoproteins, ICU, Outcome

## Abstract

**Background:**

The protective cardiovascular effect of high-density lipoproteins (HDLs) is considered to chiefly rely on reverse cholesterol transport from peripheral tissues back to the liver. However, HDL particles display pleiotropic properties, including anti-inflammatory, anti-apoptotic or antioxidant functions. Some studies suggest that HDL concentration decreases during sepsis, and an association was reported between low HDL levels and a poor outcome. Like sepsis, trauma is also associated with a systemic inflammatory response syndrome. However, no study has yet explored changes in lipid profiles during trauma. We sought to compare lipid profiles between sepsis and trauma patients in intensive care unit (ICU). In septic patients, we analyzed the association between lipid profile, severity and prognosis.

**Methods:**

A prospective, observational, single-centered study was conducted in a surgical ICU. For each patient, total cholesterol, HDL, triglyceride and low-density lipoprotein cholesterol levels were assessed at admission. Short-term prognosis outcome was prospectively assessed.

**Results:**

Seventy-five consecutive patients were admitted (50 sepsis and 25 trauma). There was no difference in SOFA and SAPSII scores between the two groups. Patients with sepsis had lower total cholesterol levels than patients with trauma. Regarding the lipoprotein profile, only HDLs differed significantly between the two groups (median [IQR] = 0.33 mmol/l [0.17–0.78] in sepsis patients versus median [IQR] = 0.99 mmol/l [0.74–1.28] in trauma patients; *P* < 0.0001). Whereas ICU mortality was not associated with lipid levels in the sepsis group, a significant negative correlation was found between HDL concentration and the length of ICU stay (*r* = −0.35; *P* = 0.03) in the group of survivor septic patients at ICU discharge. In addition, poor outcome defined as death or a SOFA score >6 at day 3 was associated with lower HDL levels (median [IQR] = 0.20 mmol/l [0.11–0.41] vs. 0.35 mmol/l [0.19–0.86] in patients with poor outcome versus others; *P* = 0.03).

**Conclusions:**

Lipid profile was totally different between sepsis and trauma in ICU patients: HDL levels were low in septic patients, whereas their concentration was not altered in trauma patients. This major difference reinforces the necessity to explore the therapeutic potential of HDL in sepsis.

**Electronic supplementary material:**

The online version of this article (doi:10.1186/s13613-017-0284-3) contains supplementary material, which is available to authorized users.

## Background

Sepsis remains an important cause of mortality in intensive care unit (ICU) despite a better comprehension of its pathophysiology [1, 2]. Sepsis pathways are complex, including early activation of pro- and anti-inflammatory signaling, along with major non-immunological responses such as cardiovascular, neuronal, autonomic, hormonal and metabolic responses, as well as activation of coagulation [3].

High-density lipoproteins (HDLs) represent a family of particles characterized by their ability to transport cholesterol from peripheral tissues back to the liver that confers to them a cardiovascular protective effect [4, 5]. HDLs also appear to emerge as relevant players in both innate and adaptive immunity [6]. Because of their pleiotropic properties, including anti-inflammatory, anti-apoptotic or antioxidant functions, experimental studies have tested the efficacy of both reconstituted HDL and ApoA1 mimetic peptide perfusion in animal models of septic shock [7–9]. Some of these have demonstrated a protective effect of these HDL mimetics on mortality and shown a decrease in the inflammatory state [9–12]. Several clinical studies have been conducted to assess HDL concentration in septic conditions. Van Leeuwen et al. [13] underlined that in septic patients, HDL concentrations rapidly fall and can be reduced to 50% of recovery conditions. Chien et al. [14] have shown that low HDL levels at day 1 of severe sepsis were significantly associated with an increase in mortality and adverse clinical outcomes. In an observational study involving 151 consecutive septic patients, a low HDL concentration was independently related to 30-day mortality [15].

In trauma patients, a systemic inflammatory response syndrome (SIRS) results from several mechanisms such as neutrophil stimulation [16] or the release of endogenous factors termed damage-associated molecular patterns (DAMPs) after tissue injury [17] leading to pro- and anti-inflammatory cytokine over-expression [18].

Whereas HDL levels were shown to be decreased during sepsis, to our knowledge, no study has explored the lipid profiles during severe injury.

The aim of the present study was to compare the lipid profiles between trauma and septic patients in ICU and to characterize the association between lipid profile, severity and prognosis.

## Methods

### Study design and patient selection

This was a prospective, observational, single-centered study conducted on 75 consecutive patients admitted to a surgical ICU of a 1000-bed, tertiary-level university hospital. Patients were recruited from July to September 2014. Criteria of inclusion were all adult trauma patients who had at least two SIRS criteria and all patients admitted to the ICU for severe sepsis or septic shock according to the Surviving Sepsis Campaign international guidelines [1]. Patients admitted with liver cirrhosis and who were immunocompromised (acquired immune deficiency syndrome, neutropenia of <1000 cells/ml or transplant surgery) were excluded from our study.

The study was approved by our local ethics committee (comité de protection des personnes de l’université Paris VII n° SC 13-026, ID RCB: 2013-A0141837), which waived the need for written informed consent because of the observational nature of the study.

### Data collection

Patient demographics, previous medical history and ICU admission diagnosis, Simplified Acute Physiology Score II (SAPSII), Sepsis-related Organ Failure Assessment score (SOFA), Injury Severity Score (ISS) for trauma patients and clinical outcomes were prospectively collected [19–21]. In case of sepsis, data on infection sites and on the type of bacteria were also collected.

For each patient, blood samples were collected at admission to the ICU to assess total cholesterol, HDL cholesterol (HDL-C), triglyceride (TG), low-density lipoprotein cholesterol (LDL-C) levels, leukocyte count, platelets, hematocrits, protein, creatinine and serum lactate. Analyses were performed in the biochemistry laboratory of the hospital. Concentrations of total cholesterol, triglycerides and HDL cholesterol were determined using routine enzymatic methods on a Bayer ADVIA 1650 analyzer, and LDL cholesterol was calculated according to the Friedewald equation. The normal range in our hospital was: LDL-C: N < 4.2 mmol/l; HDL-C: 1.0 < N < 2.0 mmol/l; total cholesterol: 3.4 < N < 6.2 mmol/l; and triglyceride 0.45 < N < 1.70 mmol/l.

ICU mortality, duration of mechanical ventilation, length of stay in ICU and in hospital, need for dialysis and vasopressor therapy, and SOFA score at day 3 and day 7 were recorded as short-term prognosis outcomes. A patient with poor outcome was defined as dead or alive with a SOFA score >6 at day 3.

### Statistical analysis

Qualitative variables are expressed as numbers (percentages), and quantitative variables are expressed as mean ± standard deviation or medians [interquartile range (IQR)] in the case of non-Gaussian distribution. Normality of distribution was checked graphically and by using the Shapiro–Wilk test.

Bivariate comparisons between the two study groups (sepsis vs trauma) were made using the Chi-square test (Fisher’s exact test was used when the expected cell frequency was <5) for qualitative variables and the Student’s *t* test for quantitative variables (or Mann–Whitney *U* test for non-Gaussian distribution). Between-group comparisons in lipid levels were further adjusted for age and sex using nonparametric analysis of covariance [22]. Given the large differences in age between the two study groups, we performed a sensitivity analysis restricted to age (±5 years)- and sex-matched patients of the two groups using Wilcoxon’s signed-rank test.

In the subgroup of patients with sepsis, we investigated the association of lipid levels with sepsis severity at ICU admission (admission SOFA score, need for mechanical ventilation and its duration) and with sepsis short-term prognosis outcomes (mortality in ICU, length of stay in ICU, poor outcome (SOFA score >6 or death) at day 3 and day 7) after adjustment for age and sex. We also investigated the association of lipid levels with bacterial species (gram-negative bacteria and gram-positive cocci) in the subgroup of septic patients. In the trauma group, we investigated the association of lipid levels with admission SOFA score, mechanical ventilation use and its duration, mortality in ICU and length of ICU stay. We used nonparametric covariance analysis to study the association of lipid levels with qualitative severity/outcome variables and partial Spearman’s rank coefficient correlation to study the association of lipid levels with quantitative severity/outcome variables.

Given the small sample size and exploratory nature of the present study, we did not adjust for multiple comparisons. All statistical tests were performed at the two-tailed α level of 0.05, and data were analyzed using the SAS software package, release 9.4 (SAS Institute, Cary, NC).

## Results

### Lipid profile in trauma and septic patients

Of the 75 patients included in the present study, 50 were admitted for sepsis and 25 for trauma. As shown in Table 1, trauma patients were younger than septic patients (*P* < 0.0001). There was no difference in SOFA and SAPSII scores between the two groups. Peritonitis was the most frequent source of sepsis infection (*n* = 22, 44%), followed by pyelonephritis (*n* = 11, 22%), angiocholitis (*n* = 9, 18%) and other sources (*n* = 8, 16%). Among septic patients, 14 of them (28%) were admitted for sepsis and 36 (72%) for septic shock according to the recent Sepsis-3 definition [2].

**Table 1:** Main patient characteristics

Characteristics	Overall (*n* = 75)	Sepsis (*n* = 50)	Trauma (*n* = 25)	P*
Age (years)	62 [45–74]	68 [57–78]	38 [28–56]	<0.0001
Male	53 (70.7)	36 (72.0)	17 (68.0)	0.72
Weight (kg)	77.3 ± 16.0	76.0 ± 17.3	80.0 ± 13.0	0.31
Lipid medications^a^	7 (9.3)	6 (12.0)	1 (4.0)	0.41
ISS	–	–	29 [20–41]	–
SAPSII	40 [29–56]	42 [30–59]	39 [26–45]	0.31
SOFA day 1	7 [5–8]	7 [4–9]	7 [5–8]	0.89
Norepinephrine day 1 (µg/kg/min)	0.1 [0.0–0.4]	0.1 [0.0–0.4]	0.1 [0.0–0.3]	0.83
Glasgow score day 1	14 [13–15]	15 [14–15]	13 [7–15]	0.0033
Leukocytes/mm^3^ day 1	14,000 [7180–21,280]	13,920 [7350–22,350]	14,250 [7180–19,200]	0.54
Hematocrit day 1 (%)	33 ± 7	33 ± 7	33 ± 7	0.83
Creatinine day 1 (mmol/l)	98 [69–136]	102 [71–163]	92 [63–99]	0.13
Protein (day 1 g/l)	54 ± 12	54 ± 13	54 ± 10	0.84
Lactates day 1 (mmol/l)	1.9 [1.3–2.3]	1.9 [1.3–2.6]	2.4 [1.3–3.2]	0.49

Values are frequencies (percentages), mean ± SD or medians [IQR]

* *P* value for comparison between sepsis and trauma patients

^a^All these patients received statins

**Fig. 1:**
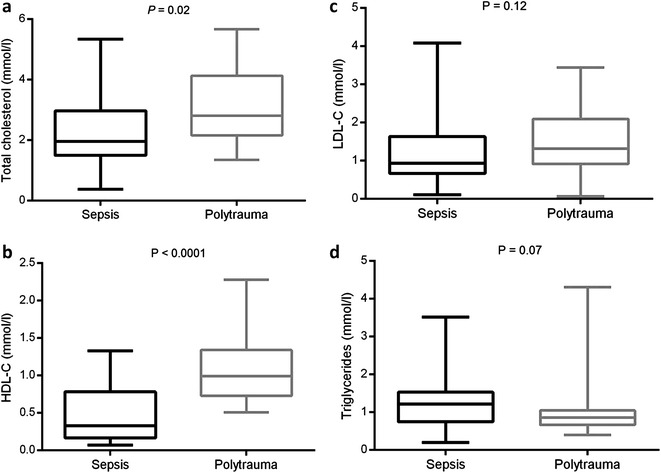
Comparison of lipid levels between sepsis and trauma patients. **a** Total cholesterol; **b** HDL-C; **c** LDL-C; **d** triglycerides. Age-/sex-adjusted *P* values were reported. Minimum, 25th, 50th, 75th percentiles and maximum change values are plotted

As shown in Fig. 1, sepsis patients had lower total cholesterol levels than trauma patients. Regarding the lipoprotein profile, only HDL-C differed significantly between the two groups. Patients with sepsis were characterized by lower HDL-C levels (median [IQR] = 0.33 mmol/l [0.17–0.78]) relative to trauma patients (median [IQR] = 0.99 mmol/l [0.74–1.28]; age- and sex-adjusted *P* < 0.0001). In sensitivity analysis restricted to age-/sex-matched patients of the two groups, a similar difference in HDL-C levels was found (median [IQR] = 0.19 mmol/l [0.14–0.92] vs. 0.98 mmol/l [0.70–1.30]; *P* = 0.001). These results are presented in Additional file 1: Table S1 in the online supplement information.

### Lipid profile and sepsis severity

In septic patients, the severity of the disease (assessed by SOFA score or the need for mechanical ventilation) was negatively associated with HDL-C, whereas no significant association was found with LDL-C and triglyceride levels (Table 2). We found a negative correlation between admission SOFA score and HDL-C levels (*r* = −0.50, *P* = 0.0004), and lower HDL-C levels in mechanically ventilated patients (median [IQR] = 0.21 mmol/l [0.13–0.45] versus 0.52 mmol/l [0.25–0.96] in patients not requiring mechanical ventilation; *P* = 0.029).

**Table 2:** Association of lipid levels with sepsis severity and short-term prognosis

	TC		LDL-C		HDL-C		Triglycerides	
	Values	P*	Values	P*	Values	P*	Values	P*
*Severity variables*								
SOFA score at day 1^2^	−0.31	0.032	0.23	0.11	−0.50	0.0004	0.14	0.34
Mechanical ventilation use^1^								
No (*n* = 23)	2.03 [1.53–3.56]	0.17	0.97 [0.66–2.20]	0.29	0.52 [0.25–0.96]	0.029	1.11 [0.79–1.32]	0.45
Yes (*n* = 27)	1.64 [1.38–2.55]		0.88 [0.68–1.36]		0.21 [0.13–0.45]		1.25 [0.62–1.57]	
Length of mechanical ventilation^2^	−0.20	0.17	−0.13	0.39	−0.39	0.006	0.23	0.11
*Bacterial species*								
Gram-negative bacteria^1^								
No (*n* = 10)	2.04 [1.64–3.74]	0.55	1.11 [0.71–1.97]	0.88	0.48 [0.19–0.96]	0.53	1.37 [1.03–2.14]	0.68
Yes (*n* = 35)	2.03 [1.29–2.83]		0.95 [0.63–1.61]		0.30 [0.15–0.62]		1.25 [0.82–1.53]	
Gram-positive cocci^1^								
No (*n* = 25)	1.81 [1.28–2.83]	0.37	0.93 [0.66–1.70]	0.83	0.21 (0.15–0.37]	0.013	1.32 [0.82–1.57]	0.57
Yes (*n* = 20)	2.07 [1.64–3.24]		0.97 [0.71–1.56]		0.61 [0.25–1.01]		1.24 [0.90–1.41]	
*Prognosis variables*								
Mortality in ICU^1^								
No (*n* = 40)	2.11 [1.56–3.17]	0.28	0.96 [0.70–1.66]	0.44	0.35 [0.17–0.83]	0.68	1.20 [0.81–1.56]	0.26
Yes (*n* = 10)	1.67 [1.26–2.11]		0.78 [0.42–1.36]		0.21 [0.17–0.55]		1.22 [0.44–1.32]	
Length of stay in ICU^2^	−0.21	0.21	−0.31	0.055	−0.35	0.030	0.21	0.20
SOFA score >6 or died at day 3^1^								
No (*n* = 29)	2.40 [1.60–3.19]	0.003	1.03 [0.68–1.86]	0.013	0.35 [0.19–0.86]	0.033	1.25 [0.88–1.53]	0.078
Yes (*n* = 15)	1.53 [1.09–2.11]		0.77 [0.42–0.99]		0.20 [0.11–0.41]		0.79 [0.44-1.32]	
SOFA score >6 or died at day 7^1^								
No (*n* = 15)	1.64 [1.52–2.55]	0.43	0.85 [0.67–1.03]	0.68	0.30 [0.11–0.78]	0.20	1.09 [0.79–1.57]	0.93
Yes (*n* = 13)	1.62 [1.28–2.11]		0.87 [0.42–1.26]		0.17 [0.12–0.37]		1.25 [0.61–1.53]	

Values are medians of lipid levels reported in mmol/l [IQR] or partial Spearman’s correlation coefficient

* Age-/sex-adjusted *P* values (^1^calculated using nonparametric ANCOVA; ^2^calculated using partial Spearman’s rank correlation)

### Lipid profile and sepsis outcome

Whereas ICU mortality was not associated with lipid levels in the sepsis group, (Table 2), a significant negative correlation was found between HDL-C and the length of ICU stay (*r* = −0.35; *P* = 0.03) in the group of survivor patients at ICU discharge.

In addition, poor outcome, defined as mortality or a SOFA score >6 at day 3, was associated with low HDL-C levels (median [IQR] = 0.20 mmol/l [0.11–0.41] vs. 0.35 mmol/l [0.19–0.86] in patients with poor outcome vs. others; *P* = 0.03).

### Lipid profile and bacterial species

In the subgroup of sepsis patients, no association was found between gram-negative bacteria and lipid levels (Table 2), whereas the presence of gram-positive cocci was associated with higher HDL-C levels (median [IQR] = 0.61 mmol/l [0.25–1.01] in patients with vs. 0.21 [0.15–0.37] in patients without; *P* = 0.013).

### Lipid profile in the subgroup of trauma patients

With the exception of the length of ICU stay, we found no statistical link between lipid profile and trauma patient characteristics. These results are presented in Additional file 2: Table S2 in the online supplement information.

## Discussion

In this study, we report that:

1. HDL-C levels were markedly low during sepsis, whereas no change was observed in the early phase of trauma relative to standard HDL concentrations.
2. Concerning LDL and TG levels, no difference was observed between sepsis and trauma.
3. In the sepsis subgroup, SOFA score, the need for mechanical ventilation and length of ICU stay were negatively associated with HDL-C levels at admission. No association between mortality and HDL-C levels was observed.

During sepsis, the dramatic decrease in HDL-C levels relative to normal conditions remains unexplained. Several hypotheses are possible, including an acute over-consumption of HDL particles, a decrease in liver HDL synthesis (especially in the case of hepatic failure) or an increased clearance following an upregulation of scavenger receptors such as SRB-1 [23]. HDL particles may also easily be redistributed from the intravascular to the extravascular compartment in the context of sepsis [13, 24]. One major action of HDL particles during sepsis is the clearance of bacterial components, such as lipopolysaccharides (LPS) [25]. After binding bacterial components, the clearance of HDL particles may also be increased in inflammatory conditions [25, 26]. Interestingly, in the population of septic patients studied here, HDL concentration was higher in patients with gram-positive versus gram-negative bacteria, suggesting that the clearance of HDL-LPS particles may be enhanced.

Both sepsis and trauma are characterized by an intense systemic inflammation that results from a disproportionate activation of inflammation signaling pathways [16, 27, 28]. In these two pathologies, cell injury initiates the secretion of different mediators such as DAMPs, which are recognized by the host, leading to a pro-inflammatory response [3, 29]. Also, similar leukocyte genomic responses to inflammation may be involved in both trauma and sepsis patients [30]. However, despite a common inflammatory background, we did not observe a similar lipid profile between sepsis and trauma patients. To our knowledge, except in spinal cord injury [31], lipid profile and especially HDL-C concentration have never been compared between trauma and septic patients. In this context, we show, for the first time, that in the early phase of trauma, HDL levels are preserved, contrasting with sepsis. The mechanisms that may explain the HDL-C level differences between trauma and sepsis remain unknown, but the interaction between HDL particles and LPS may be one of the causes [23]. The marked release of bacterial endotoxins may lead to an increased consumption of HDL particles.

This discrepancy between sepsis and trauma patients emphasizes the potential protective role of HDLs during sepsis. In view of HDL over-consumption and clearance in sepsis, their previously described pleiotropic effects may be lacking for the control of inflammation associated with this condition [7, 23].

Concerning the relationship between HDL-C levels and prognosis in septic patients, results of previous studies are controversial. On the one hand, Chien et al. [14] have shown that HDL-C levels in non-survivors were significantly lower than those of survivors from day 1 to day 4 and that HDL-C concentration at day 1 could predict the overall 30-day mortality rate. In their study, the cutoff value of 0.52 mmol/l had a sensitivity of 92% and a specificity of 80% for predicting the overall 30-day mortality rate. Barlage et al., in an observational study in ICU, also underlined the relationship between apo-A1 levels and 30-day mortality [15]. On the other hand, Lee et al. [32] reported no significant HDL-C differences between survivors and non-survivors in 117 septic patients. Only TG levels were associated with mortality.

Our study was not designed to test a potential difference in mortality but rather to compare trauma and septic patients. Comparing with the studies of Chien et al. and Barlage et al., the limited number of patients and the low proportion of sepsis in our cohort may explain this lack of relationship between HDL concentration and mortality. However, we found that death or a SOFA score >6 at day 3 was associated with low HDL-C levels. To our knowledge, only one previous study has explored the relationship between HDL concentrations at admission and organ failure progression: Cirstea et al. [33] have recently reported that low HDL-C levels at the time of admission for suspected sepsis were strongly and independently prognostic of subsequent multiple organ dysfunction.

One major finding of our study was that SOFA score, need for and duration of mechanical ventilation and length of ICU stay were all negatively associated with HDL-C levels. This result concerning length of ICU stay was similar to that found by Chien and al. [14]. Concerning SOFA score evaluation at day 1, our results are in line with those of Ferreira et al., showing that the sequential assessment of organ dysfunction during the first days of ICU admission was clinically relevant and a good indicator of prognosis [34].

Our study has some limitations. The first limitation is the marked difference in age between sepsis and trauma groups. A sensitivity analysis restricted to age-/sex-matched patients from the two groups was performed, and the lower levels in HDL-C in sepsis relative to trauma patients were confirmed. In addition, most cross-sectional studies in the general population report no difference in HDL levels between young and older subjects [35–38]. Taken together, age adjustment, sensitivity analysis and bibliographic data support our conclusion that HDL level differences are indeed due to septic conditions.

Second, we were not allowed to collect information on race and familial disorders of lipid metabolism which can affect the lipid levels.

Third, we have not measured inflammatory markers such as cytokines/chemokines in the two groups of patients. In this context, it is not possible to compare the inflammatory state between trauma and septic patients. However, SAPSII and SOFA scores were collected and no differences were found between trauma and septic patients, suggesting that these two groups of patients were comparable in terms of gravity.

Fourth, we analyzed lipid levels only at ICU admission. No additional measures were performed in the early phase of sepsis (i.e., at emergency room admission) and during the stay in ICU. Comparison between the basal lipid levels (i.e., without any episode of sepsis or trauma before admission to hospital) and the pathologic condition in the same patient would be of interest.

Fifth, peritonitis, urinary tract infection and angiocholitis are the main causes of sepsis recruitment in our surgical ICU, which is quite different from other ICUs and especially medical ICUs, which may explain some differences. The ability of HDL to bind and promote LPS clearance in cases of infection by gram-negative pathogens may also impact our results because our sepsis population involved more gram-negative pathogens.

Finally, additional studies with appropriate sample size calculation should be performed, with a larger number of patients. Our study is exploratory, with a limited sample size; our conclusion should therefore be taken with caution.

Regarding the main finding of our study, further experimental studies should be conducted to understand the reasons for the low HDL concentration during sepsis. In addition, a lipidomics approach may also be of interest. Dysfunctional HDLs during sepsis may represent an explanation for the observed organ dysfunction during sepsis [23]. The interaction between HDL particles and bacterial components, such as LPS, should also be addressed in future experimental studies. Finally, because of the pleiotropic effects of HDLs, therapeutic injection of HDL mimetic peptides or reconstituted HDLs may be of interest and should be tested in preclinical settings.

## Conclusion

Our study has demonstrated that the lipid profile was different between sepsis and trauma ICU patients. HDL-C levels were low in septic patients, whereas their concentration was not altered in the case of trauma. This major difference reinforces the necessity to explore the therapeutic potential of HDL in sepsis.

In the subgroup of sepsis, SOFA score, need for and duration of mechanical ventilation and length of ICU stay were all negatively associated with HDL-C levels.

Our study suggests that HDLs may play an important role in sepsis. Further studies enrolling more patients, and especially septic patients, are necessary to test whether low HDL levels are a bystander prognosis marker of mortality or whether HDLs may limit the deleterious effects of sepsis.

## Additional files

**Additional file 1.** Sensitivity analysis in age/sex-matched patients from the two groups.

**Additional file 2.** Association of lipid levels with trauma severity and short-term prognosis.

**Additional file 3.** Manuscript database.

## Abbreviations

DAMPs: damage-associated molecular patterns
HDL: high-density lipoprotein
ICU: intensive care unit
ISS: injury severity score
LPS: lipopolysaccharides
LDL: low-density lipoprotein cholesterol
SOFA: Sepsis-related Organ Failure Assessment Score
SAPSII: Simplified Acute Physiology Score II
SIRS: systemic inflammatory response syndrome
TG: triglyceride
