# Supplementary material for: Low HDL levels in sepsis versus trauma patients in intensive care unit
Source: Ann Intensive Care. 2017 Jun 6;7:60. doi: 10.1186/s13613-017-0284-3 (PMC5461227; doi:10.1186/s13613-017-0284-3)
Supplement: Supplementary file 1 — Additional file 1. Sensitivity analysis in age/sex-matched patients from the two groups. [file 13613_2017_284_MOESM1_ESM.docx]

**Table S1: sensitivity analysis in age/sex-matched patients**

| **Characteristics** | **Sepsis (n=16)** | **Trauma (n=16)** | ***P **** |
| --- | --- | --- | --- |
| Age, years | 55 [42-64] | 54 [43-62] | 0.94 |
| Male | 8 (50.0) | 8 (50.0) | - |
| Weight, kg | 66.5 [60.0-77.0] | 88.5 [65.0-91.5] | 0.047 |
| ISS | - | 29.0 [20.5-38.5] | - |
| SAPSII | 37 [23-57] | 40 [32-57] | 0.35 |
| SOFA day1 | 7 [5-9] | 8 [6-9] | 0.63 |
| Norepinephrine day 1, µg/kg/min | 0.3 [0.0-0.4] | 0.1 [0.0-0.6] | 1.00 |
| Glasgow score day 1 | 15 [14-15] | 13 [7-15] | 0.017 |
| Leucocytes/mm3 day 1 | 17180 [9540-22520] | 14210 [7165-18465] | 0.53 |
| Hematocrit day 1, % | 33 [28-35] | 34 [29-37] | 0.71 |
| Creatininemia day 1, mmol/l | 80 [65-132] | 95 [67-104] | 0.64 |
| Protein, day 1 g/L | 56 [49-66] | 53 [45-57] | 0.25 |
| Lactates day 1, mmol/l | 1.8 [1.3-2.2] | 2.8 [1.2-3.6] | 0.25 |
| TC, mmol/l | 1.72 [1.51-3.53] | 2.95 [2.24-4.13] | 0.065 |
| HDL-C, mmol/l | 0.19 [0.14-0.92] | 0.98 [0.70-1.30] | 0.001 |
| LDL-C, mmol/l | 0.90 [0.57-1.92] | 1.48 [1.08-2.16] | 0.23 |
| Triglycerides, mmol/l | 1.40 [1.14-1.85] | 0.87 [0.77-1.03] | 0.009 |

Values are frequencies (percentages) or medians [IQR].

*P-Value for comparison between sepsis and trauma patients calculated using Wilcoxon signed rank test.
