# Supplementary material for: Low HDL levels in sepsis versus trauma patients in intensive care unit
Source: Ann Intensive Care. 2017 Jun 6;7:60. doi: 10.1186/s13613-017-0284-3 (PMC5461227; doi:10.1186/s13613-017-0284-3)
Supplement: Supplementary file 2 — Additional file 2. Association of lipid levels with trauma severity and short-term prognosis. [file 13613_2017_284_MOESM2_ESM.docx]

**Table S2. Association of lipid levels with trauma severity and short-term prognosis**

|  | **TC** | | **LDL-C** | | **HDL-C** | | **Triglycerides** | |
| --- | --- | --- | --- | --- | --- | --- | --- | --- |
|  | Values | *P** | Values | *P** | Values | *P** | Values | *P** |
| SOFA score at day 1 ^2^ | -0.34 | 0.11 | -0.24 | 0.29 | -0.37 | 0.085 | 0.07 | 0.75 |
| Mechanical ventilation use ^1^ |  |  |  |  |  |  |  |  |
| No (n=5) | 3.39 [2.48-4.78] | - | 1.82 [0.98-2.20] | - | 1.03 [0.98-1.40] | - | 0.86 [0.66-1.02] | - |
| Yes (n=20) | 2.70 [2.16-3.58] |  | 1.20 [0.87-2.04] |  | 0.99 [0.70-1.23] |  | 0.84 [0.71-1.05] |  |
| Length of mechanical ventilation ^2^ | -0.25 | 0.25 | -0.23 | 0.30 | -0.22 | 0.31 | 0.29 | 0.17 |
| Mortality in ICU ^1^ |  |  |  |  |  |  |  |  |
| No (n=23) | 2.81 [2.19-3.82] | - | 1.32 [0.98-2.04] | - | 0.99 [0.74-1.28] | - | 0.81 [0.66-1.02] | - |
| Yes (n=2) | 1.35-4.44 |  | 0.07-2.11 |  | 0.56-1.87 |  | 1.03-1.59 |  |
| Length of stay in ICU ^2^ | -0.60 | 0.004 | -0.46 | 0.042 | -0.52 | 0.016 | 0.05 | 0.85 |

Values are median of lipid levels reported in mmol/l [IQR] or partial Spearman’s correlation coefficients.

* age-sex-adjusted P *V*alues (^1^ no comparison because of small number of events (≤5); ^2^ calculated using partial Spearman’s rank correlation)
